# Penile Urethral Stricture After Radical Prostatectomy – Use of Aldehyde-Free Bovine Pericardium Graft

**DOI:** 10.1590/S1677-5538.IBJU.2026.9906

**Published:** 2026-04-28

**Authors:** Nathália P. S. Mello, Rodrigo R. Vieiralves, Arthur M. Gomes, José A. D. Resende, Luciano A. Favorito

**Affiliations:** 1 Hospital Federal da Lagoa Serviço de Urologia Rio de Janeiro RJ Brasil Serviço de Urologia, Hospital Federal da Lagoa, Rio de Janeiro, RJ, Brasil

## Abstract

**Objective:**

Penile urethral stricture is a potential complication after any form of urethral instrumentation. Treatment options may be limited when considering direct urethrotomies or urethroplasties without grafting, due to the increased risk of penile shortening and functional compromise. In this context, among the various graft materials described in the literature (including buccal mucosa, lingual mucosa, bladder mucosa, colonic mucosa, augmentation urethroplasty with skin flaps, and injectable antifibrotic agents) (1, 2) the use of bovine pericardium appears promising (3).

In this video, we present a clinical case in which the L-Hydro® tissue treatment technology 100% aldehyde free, VIVENDI™ graft was used as a graft for the surgical repair of penile urethral stricture following radical prostatectomy.

**Materials and Methods:**

The present study was approved by the hospital's Institutional Ethics Committee in accordance with ethical standards for research involving human subjects. A 65-year-old male patient developed a penile urethral stricture following urethral instrumentation and prolonged urinary catheter use after radical prostatectomy. Preoperative evaluation included cystourethrography, which demonstrated a 1.2 cm stricture in the penile urethra.

Urethroplasty was indicated for definitive surgical management. Under regional anesthesia, a longitudinal penile incision was made, followed by a ventral sagittal urethrotomy directly over the stricture segment. A free aldehyde-free bovine pericardium graft (VIVENDI™) was tailored to the defect and placed as a dorsal onlay within the urethrotomy. The graft was secured with interrupted 4-0 poliglecaprone 25 sutures. The ventral urethrotomy was closed over a 16 Fr silicone Foley catheter, and the penile incision was closed in layers. Follow-up assessments included uroflowmetry and post-void residual urine measurement at 4 weeks, with urethroscopy performed at 7 weeks postoperatively to evaluate urethral patency and graft integration.

**Results:**

No intraoperative or postoperative complications occurred. Seven weeks after surgery, the patient underwent urethroscopy, which demonstrated a well-appearing urethral lumen without evidence of stricture or signs of infection. The graft was observed to be well incorporated into the urethral wall with no adverse tissue reaction. At follow-up, the patient also demonstrated adequate bladder emptying, with satisfactory voiding parameters and low post-void residual urine.

**Conclusions:**

In this reported case, the use of a bovine pericardium graft demonstrated satisfactory results in penile urethroplasty for the treatment of a urethral stricture. Based on current evidence, bovine pericardium appears to be a feasible graft option for complex urethral reconstruction. However, further studies involving larger patient cohorts and multicenter collaboration are necessary to confirm these findings and better define the long-term efficacy and safety of this approach.

**Available at:**VIDEO

## Data Availability

Data Not Provided
